# Recovery After Stroke: New Insight to Promote Brain Plasticity

**DOI:** 10.3389/fneur.2021.768958

**Published:** 2021-11-19

**Authors:** Laura Otero-Ortega, María Gutiérrez-Fernández, Exuperio Díez-Tejedor

**Affiliations:** Neurological Sciences and Cerebrovascular Research Laboratory, Department of Neurology and Stroke Center, La Paz University Hospital, Neuroscience Area of Hospital La Paz Institute for Health Research (IdiPAZ), Universidad Autónoma de Madrid, Madrid, Spain

**Keywords:** brain plasticity, recovery, stroke, trophic factors, stem cell, extracellular vesicles

## Introduction

The recovery of the patient is the goal of the neuroscientists after a stroke. To achieve this recovery, there is a crucial need for increasing the understanding of the pathophysiological mechanisms that spontaneously engage early after stroke, which involve excitotoxicity, free radical damage, increased glutamate concentrations, and inflammation, leading to cell death. To assume that only neurons are vulnerable to these pathophysiological responses is simplistic. Stroke affects all components of the neurovascular unit, which consists of endothelial cells, pericytes, neurons, glial cells, white matter fiber tracts, myelin, and extracellular matrix proteins. It is, therefore, important to focus on brain protection as a whole rather than neuroprotection in isolation (1).

**Figure 1:**
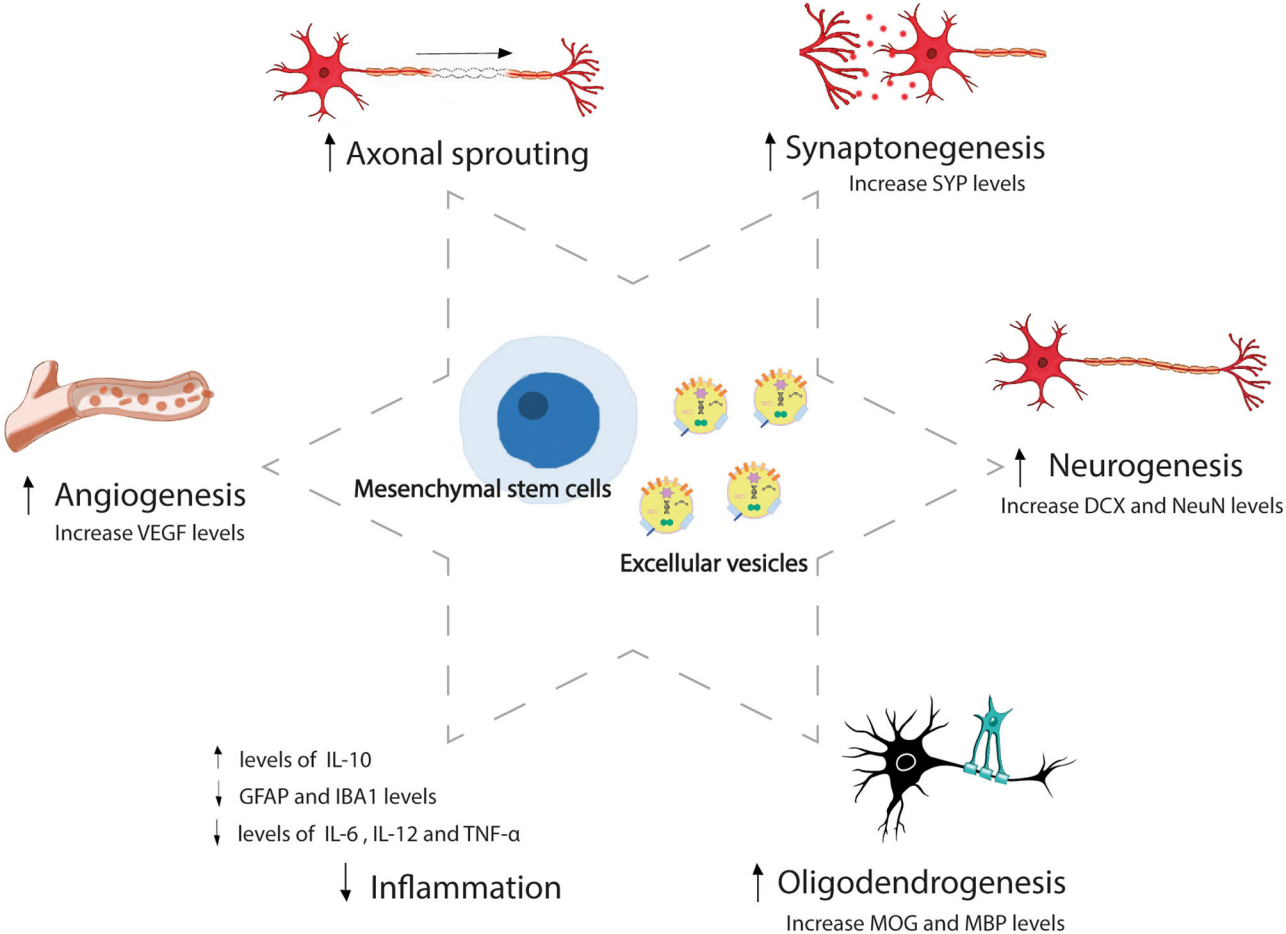
Mesenchymal stem cell and extracellular vesicles-induced brain repair process after stroke. Mesenchymal stem cells and extracellular vesicles administration-induced brain plasticity that involves adaptive structural and functional changes, including angiogenesis, axonal sprouting, synaptogenesis, neurogenesis, oligodendrogenesis, and inflammation modulation after stroke. SYP, synaptophysin; DCX, doublecortin; NeuN, neuronal nuclear protein; MOG, myelin-oligodendrocyte glycoprotein; MBP, myelin basic protein; IL, interleukin; GFAP, glial fibrillary acidic protein; IBA, ionized calcium-binding adapter molecule; TNF, tumor necrosis factor; VEGF, vascular endothelial growth factor.

In the last 50 years, we have gained significant insights into the molecular mechanisms of recovery after stroke, in addition to the damage mechanisms. Due to its plasticity, the brain has the ability to reorganize its function and structure, which involves processes of self-protection and self-repair. Brain plasticity is a very complex process that involves adaptive structural and functional changes in the brain, including neurogenesis, synaptogenesis, angiogenesis, oligodendrogenesis, and astrogliosis modulation, and promotes collateral circulation, processes that begin immediately after stroke (2–4). Such is the self-repair ability brain that the stroke-induced neurogenesis is not only limited to the subventricular zone and hippocampal dentate gyrus. It has also been revealed that several additional areas of the brain promote mammalian adult neurogenesis, which include the hypothalamus, striatum, substantia nigra, cortex, and amygdala (5). The neural stem cells of the neurogenic areas generate new neural cells that migrate to the lesion site and become mature neurons, orchestrating neurological repair through nerve repair, neuron polarization, axonal sprouting and pruning, neurite outgrowth, and myelin repair, promoting post-stroke recovery (4). However, in the unfavorable microenvironment that occurs in the lesion, most new cells do not survive (4). Given that this self-repair capacity is limited, there has been a growing interest in the potential for brain plasticity-inducing interventions to enhance post-stroke recovery through rehabilitation, trophic factors, cell therapy, and extracellular vesicles. These therapeutic approaches currently hold great promise by targeting the mechanisms involved in brain plasticity (2, 6–8) (Figure 1). The beneficial effects of rehabilitation therapies in stroke recovery are well-known (9), although there are still certain aspects to be clarified, such as the optimal time to start rehabilitation, as well as its intensity and duration. Other approaches that involve brain stimulation, such as transcranial magnetic and electrical stimulation, can enhance recovery and post-stroke plasticity (10), and innovations with exoskeletons and brain-machine interfaces (11) have opened up new research lines. However, we would like to focus on other novel and promising strategies, such as the administration of trophic factors, stem cells, and extracellular vesicles.

## Trophic Factors as Therapeutic Strategy to Promote Brain Plasticity

In recent years, trophic factors have generated a great deal of interest in the clinical context, given that their administration is not restricted to a narrow therapeutic window. Several trophic factors, including erythropoietin, brain-derived neurotrophic factor, granulocyte-colony-stimulating factor, vascular endothelial growth factor, fibroblast growth factor, epidermal growth factor, and heparin-binding epidermal growth factor, have anti-inflammatory and anti-excitotoxic protective properties and have demonstrated efficacy in promoting neurogenesis and angiogenesis, stimulating progenitor cell proliferation, preventing blood-brain barrier (BBB) disruption and ultimately promoting functional recovery in experimental stroke models (12–15). However, a number of these approaches were lost in translation from a bench to a bedside (14, 15), while others have not yet been tested in clinical trials.

## Cell Therapy: The Factory of Trophic Factors and Key Molecules to Improve Recovery

But why settle for administering a single factor when we can administer the whole arsenal? This is where stem cell therapy plays an important role. Stem cells can secrete various trophic factors and key molecules, promote brain plasticity, and reduce overall inflammation. In particular, mesenchymal stem cells (MSCs) from bone marrow or adipose tissue have demonstrated efficacy in experimental animal stroke models (16–23). These positive findings are translated to clinical trials, where MSCs and other cells (such as bone marrow mononuclear cells) have demonstrated safety in patients with stroke (24–30) and even efficacy in promoting improvement in white matter injuries at 1 year (31). Perhaps, one of the most stimulating findings in stem cell translational research is the discovery of an abundant quantity of MSCs within adipose tissue. Adipose tissue-derived MSCs (AD-MSCs) are of special interest, not only due to their abundance but also their relative ease of obtention through procedures such as liposuction and abdominoplasty, which obviate the ethical concerns with embryonic MSCs. Due to immunoprivileged characteristics of this cell type, allogeneic administration is possible, in our opinion (32). Treatment can, therefore, be administered at an early stage, which is crucial for a disease where delays result in irrevocable loss of brain function. The administration of AD-MSCs in the acute phase could inhibit the aforementioned pathophysiological mechanisms that are activated early after stroke, thereby participating not only in the repair processes of the neurovascular unit but also in its protection. In terms of clinical feasibility, the intravenous route for delivering AD-MSCs is attractive, given its low invasiveness, low risk, and greater comfort for the patient. Using this route, AD-MSCs are unable to reach the brain due to their lack of ability in crossing the BBB; however, they exert their beneficial therapeutic actions by delivering their secretome from the peripheral organs where they are confined (19). MSCs exert their action by the release of key molecules or extracellular vesicles (EVs) by paracrine effects (33), rather than through differentiation to replace damaged neurons (16, 18).

## Extracellular Vesicles: The Novel Strategy to Enhance Brain Recovery

Extracellular vesicles are released from all cell types, harbor important molecules such as proteins, DNA, lipids, mRNAs, and microRNAs, and participate in cell-to-cell communication. EVs can act as an active principle promoting several mechanisms of recovery after stroke, including brain plasticity. It has been shown that the intravenous administration of MSC-derived EVs promotes functional recovery and brain plasticity in an ischemic stroke rat model (34–37). Moreover, our group found that an intravenous administration of EVs-improved outcomes by promoting the processes involved in white matter repair in subcortical stroke in rats (35). Other authors have also shown that EVs induce higher axonal density and neurite remodeling (34), new formation of endothelial cells (36), sprouting of new capillaries, and higher endothelial integrity (37). MSC-derived EVs are, therefore, a promising approach for repairing the components of the neurovascular unit to promote overall post-stroke recovery (38, 39).

MSC-derived EVs are able to go one step farther than MSCs as a therapeutic strategy, given that MSC-EVs can cross the BBB, resolve cell-related problems, such as immune compatibility, tumor formation, and vascular occlusion, and can be stored in hospital settings without the need for toxic cryopreservative agents, offering an approach for acute ischemic stroke. Due to their small size, MSC-EVs can be saved from phagocytosis by macrophages. In addition, selective manipulation of their cargo by bioengineering can lead to individualized medicine (40, 41). There is, currently, only one ongoing clinical trial aimed at assaying the efficacy of the allogeneic administration of MSC-derived EVs enriched by miR-124 for improving the recovery of patients with acute ischemic stroke, registered in clinicaltrials.gov (Identifier: NCT03384433). A previous study using an animal model of stroke demonstrated that the administration of EVs loaded with miR-124 promoted cortical neural progenitors and neurogenesis (42). These functions of the microRNA content of EVs make them important not only as treatment but also as biomarkers. We showed that circulating EVs from patients with stroke contain miRNA and proteins related to risk factors and etiology, post-ischemic immune response, endogenous protection, and angiogenesis (43). We also observed differences in the levels of the microRNA content of EVs according to the topography of the stroke (subcortical and cortical-subcortical ischemic stroke) and related to improved recovery after stroke (44). Given the progress of research on EVs, further information on brain-derived EVs under stroke conditions is necessary (45). The content of EVs can be used as biomarkers that improve our understanding of the mechanisms by which EVs act in stroke to help develop new therapeutic strategies and find new molecular targets for this neurological disease.

## Discussion

The experimental studies in animal model and clinical trials have indicated that intravenously administered MSC therapy seems to be a promising therapeutic strategy after stroke (16–23, 26–28, 30). However, there are still some unresolved issues that have to be investigated such as the most appropriate administration timing, the doses required for successful recovery (46), and the dose regimen (single-dose or repeat-doses) that reach the higher threshold of brain repair after stroke. Likewise, the limitations of cell therapy will be determined by the results of clinical trials.

Moreover, MSC-derived EVs have some important advantages that can be exploited when translating a therapeutic strategy for stroke. EVs provide a great feature as a drug delivery system, given their ability to cross the BBB (40). Thanks to the development of delivery system technologies, MSC-derived EVs can be engineered and designed to carry specific therapeutic molecules (47) according to brain tissue repair needs, avoiding molecules that could induce adverse effects, moving toward personalized medicine.

Beyond the proven beneficial outcomes with the MSC-derived EV treatment in experimental animal models of stroke (34–39), many aspects are yet to be resolved about the production of EVs for their use in clinical practice, such us large-scale production, conditions in different physiologically environments, and standardized experimental protocol for extracting EVs to provide batch uniformity according to GMP regulations (40, 48, 49).

In conclusion, we are facing a disease that has a high incidence and prevalence and results in major disability. However, we are also finding new and promising therapeutic options, such as trophic factors, cell therapy, and EVs that can positively contribute to stroke recovery by improving brain plasticity. For EVs, their ability to cross the BBB and their editable cargo provide live information on molecules that participate in damage and post-stroke repair, which could lead to personalized and precision medicine.

## Author Contributions

All authors listed have made a substantial, direct, and intellectual contribution to the work, and approved it for publication.

## Funding

This work was sponsored by a grant from Miguel Servet (CP15/00069; CPII20/00002 to MG-F), Miguel Servet (CP20/00024 to LO-O), and the INVICTUS PLUS network grant (RD16/0019/0005) from the Carlos III Health Institute Health Care Research Fund and was co-funded by the European Regional Development Fund (ERDF). We appreciate Morote Traducciones S. L. support for their editing assistance.

## Conflict of Interest

The authors declare that the research was conducted in the absence of any commercial or financial relationships that could be construed as a potential conflict of interest.

## Publisher's Note

All claims expressed in this article are solely those of the authors and do not necessarily represent those of their affiliated organizations, or those of the publisher, the editors and the reviewers. Any product that may be evaluated in this article, or claim that may be made by its manufacturer, is not guaranteed or endorsed by the publisher.
